# Evaluating pathogenicity of *SLC34A3*-Ser192Leu, a frequent European missense variant in disorders of renal phosphate wasting

**DOI:** 10.1007/s00240-019-01116-2

**Published:** 2019-02-23

**Authors:** Ria Schönauer, Friederike Petzold, Wilhelmina Lucinescu, Anna Seidel, Luise Müller, Steffen Neuber, Carsten Bergmann, John A. Sayer, Andreas Werner, Jan Halbritter

**Affiliations:** 1 Division of Nephrology, Department of Internal Medicine, University Hospital Leipzig, Liebigstr. 20, 04103 Leipzig, Germany; 2 School of Biomedical Sciences, University of Newcastle, Newcastle upon Tyne, UK; 3 Bioscientia, Institute of Human Genetics, Ingelheim, Germany; 4 Institute of Genetic Medicine, International Centre for Life, University of Newcastle, Newcastle upon Tyne, UK; 5 Newcastle Hospitals NHS Foundation Trust, Freeman Road, Newcastle upon Tyne, NE7 7DN UK; 6 NIHR Newcastle Biomedical Research Centre, Newcastle upon Tyne, UK; 7 Institute for Cell and Molecular Biosciences, Epithelial Research Group, University of Newcastle, Framlington Place, Newcastle upon Tyne, NE2 4HH UK

**Keywords:** HHRH, SLC34A3, NaPi2c, Nephrocalcinosis, Nephrolithiasis, Hypophosphatemia, Hypercalciuria

## Abstract

Loss-of-function mutations of *SLC34A3* represent an established cause of a distinct renal phosphate wasting disorder termed hereditary hypophosphatemic rickets with hypercalciuria (HHRH). *SLC34A3* encodes the renal phosphate transporter NaPi2c expressed at the apical brush border of proximal renal tubules. Substitution of p.Ser192Leu is one of the most frequent genetic changes among HHRH patients in Europe, but has never been systematically evaluated, clinically or on a cellular level. Identification of a 32-year-old female with a homozgyous c.575C>T, p.Ser192Leu substitution enabled a more comprehensive assessment of the impact of this missense variant. Clinically, the patient showed renal phosphate wasting and nephrocalcinosis without any bone abnormalities. Heterozygous carriers of deleterious *SLC34A3* variants were previously described to harbor an increased risk of kidney stone formation and renal calcification. We hence examined the frequency of p.Ser192Leu variants in our adult kidney stone cohort and compared the results to clinical findings of previously published cases of both mono- and biallelic p.Ser192Leu changes. On a cellular level, p.Ser192Leu-mutated transporters localize to the plasma membrane in different cellular systems, but lead to significantly reduced transport activity of inorganic phosphate upon overexpression in *Xenopus* oocytes. Despite the reduced function in ectopic cellular systems, the clinical consequences of p.Ser192Leu may appear relatively mild, at least in our index patient, and can potentially be missed in clinical practice.

**Electronic supplementary material:**

The online version of this article (10.1007/s00240-019-01116-2) contains supplementary material, which is available to authorized users.

## Introduction

Tight regulation of serum phosphate is crucial for various cellular processes, such as maintenance of energy balance, prevention of vascular calcification, bone metabolism, and acid–base homeostasis [1]. Driven by the phosphaturic effects of parathyroid hormone (PTH) and fibroblast growth factor 23 (FGF23), the kidneys play an important role in the excretion of excess phosphate from the circulation. The phosphaturic effect is achieved by inhibition of renal phosphate reabsorption from the primary urine via two Na^+^-dependent phosphate transporters NaPi2a/SLC34A1 and NaPi2c/SLC34A3 [2]. On the other hand, low serum phosphate triggers vitamin D-dependent enteral calcium reabsorption and may lead to hypercalcemia and hypercalciuria.

Hypercalciuria is the most common metabolic finding among kidney stone formers [3]. Its genetic basis, however, is poorly understood. Monogenic kidney stone disorders shed light on mechanisms of hypercalciuria that may also pertain to the general population of kidney stone formers [4]. One of these disorders is hereditary hypophosphatemic rickets with hypercalciuria (HHRH; OMIM #241,530) characterized by renal phosphate wasting and elevated calcium levels in blood and urine. In 1987, Tieder et al. first described isolated hypercalciuria and HHRH as two phenotypic expressions of a shared underlying defect in an extended Bedouin kindred [5]. It took another 19 years until two groups independently deciphered loss-of-function mutations of the proximal renal tubular phosphate transporter SLC34A3/NaPi2c as the underlying genetic defect [6, 7]. The electroneutral transporter NaPi2c, like its electrogenic paralog NaPi2a/SLC34A1, is expressed at the luminal brush border of proximal renal tubular cells, responsible for fine-tuning phosphate reabsorption from primary urine to the basolateral bloodstream. Malfunction or loss of function of NaPi2c results in urinary phosphate wasting, hypophosphatemia and a consecutive increase in systemic 1,25-(OH)_2_D levels [8]. Among several mutations identified, both initial genetic studies found a heterozygous missense variant at amino acid residue 192 changing serine to leucine as part of compound heterozygous *SLC34A3* mutations [6, 7]. Although the p.Ser192Leu variant has been reported several times in patients with HHRH and isolated hypercalciuria, it has never been systematically evaluated with regard to clinical or molecular pathogenicity. In recessive disorders homozygous mutations are helpful to assess the clinical impact of a certain genetic variant as exemplified here by a young adult with nephrocalcinosis due to a homozygous *SLC34A3* c.575C > T, p.Ser192Leu mutation. We provide an evaluation of the mutation’s clinical and molecular impact and raise the question whether p.Ser192Leu may be enriched in idiopathic kidney stone formers.

## Materials and methods

### Patient cohort

The index patient was recruited for genetic analysis after informed consent and enrollment in our clinical registry for hereditary kidney stone disorders at the University of Leipzig (Germany) [9]. Patients with idiopathic kidney stone disease were tested for genetic causes after written informed consent and prior study approval by the Institutional Review Board (IRB) of the Universities of Leipzig and Newcastle. The cohort comprised 670 patients, 61% males and 35% females, with a median age of 50 years (range 1–88 years) and a median age at first stone event of 36 years (range 0–86 years).

### Expression vectors

The vector pEGFP_C1_NaPi2c_WT [10] for the expression of a fusion protein of N-terminal enhanced green fluorescent protein (eGFP) connected by a linker sequence (SGLRSRAQASNS) to wild-type NaPi2c was constructed by ligation of the human *SLC34A3*-sequence (NM_001177316.1) into the pEGFP-C1 vector backbone using EcoRI and SacII restriction sites that were added by mutagenesis [7]. To establish the mutant plasmid pEGFP_C1_NaPi2c_S192L, the Q5® Site-Directed Mutagenesis Kit (New England Biolabs, Ipswich, Massachusetts, USA) and custom-designed back-to-back primers (NaPi2c_S192L_Fw: 5′-GCTTTCAGCGGCTTGGCGGTGCAC-3′; NaPi2c_S192L_Rv: 5′- CCTCTGAAATTCATCCCGGTCCCCTG-3′) were used to introduce the base exchange 575C>T into pEGFP_C1_NaPi2c_WT according to the manufacturer’s instructions. Correctness of all sequences was verified by Sanger sequencing.

### Cell culture

Human embryonic kidney cells (HEK293) were cultured in a mixture of Dulbecco’s modified Eagle’s medium and Ham’s F12 (DMEM/F12, catalog number 21,331,046, Thermo Fisher Scientific, Waltham, Massachusetts, USA) supplemented with 15% fetal bovine serum (FBS Superior, Burlington, Massachusetts, USA) under humidified atmosphere at 37 °C and 5% CO_2_.

### Live cell microscopy

HEK293 cells were grown to 70–80% confluence on µ-Slide 8 Wells (ibidi, Martinsried, Germany), transfected with 1 µg pEGFP_C1_NaPi2c_WT or pEGFP_C1_NaPi2c_S192L using 1 µl Lipofectamine^®^2000 (Invitrogen, Carlsbad, USA) and incubated overnight under humidified athmosphere at 37 °C and 5% CO_**2**_. 24 h after transfection, the cells were starved in 200 µl Opti-MEM^™^ Reduced Serum Medium with GlutaMAX^™^ supplement (Thermo Fisher Scientific, Waltham, Massachusetts, USA) and membrane expression of fluorescently labeled proteins in living cells was documented using an AxioObserver.Z1 microscope with an ApoTome Imagig System (Carl Zeiss AG, Oberkochen, Germany).

### Molecular biology oocyte injections

Constructs for oocyte injections were generated by PCR adding a T7 RNA transcription start site and a short poly-A tail using mammalian SLC34A3 expression plasmids as templates [10]. The primer sequences are:

T7 – NaPi2c, 5′-AGCCACTAATACGACTCACTATAGGGCTCCGCTGCCACCATGCCGAGTTCCCTTCC-3′; T7 – GFP-NaPi2c, 5′-AGCCACTAATACGACTCACTATAGGGCTCCGCTGCCACCATGGTGAGCAAGGGCGAG-3′ and SV40 pA terminator, 5′-TTTTTTTTTTTTTTTTTTTTTTTTTTTTCAAAATATTAACGCTTACAATTTACGCG-3′. 0.1 µg of the purified DNA fragments was in vitro transcribed using the T7 mMessageMachine kit (Thermo Fisher Scientific, Waltham, Massachusetts, USA) and 10 ng of RNA was injected per oocyte.

### *Xenopus laevis* oocytes

Oocytes were purchased from Ecocyte (Dortmund, Germany) and established protocols for maintenance and injections were followed [11]. Oocytes were assayed after 3 days measuring fluorescence and uptake of [^32^P] inorganic phosphate. Fluorescence at the oocyte surface was measured using an inverted fluorescence microscope (Nikon, Tokyo, Japan) with identical settings for each measurement and quantified using image FIJI.

## Results

**Fig. 1:**
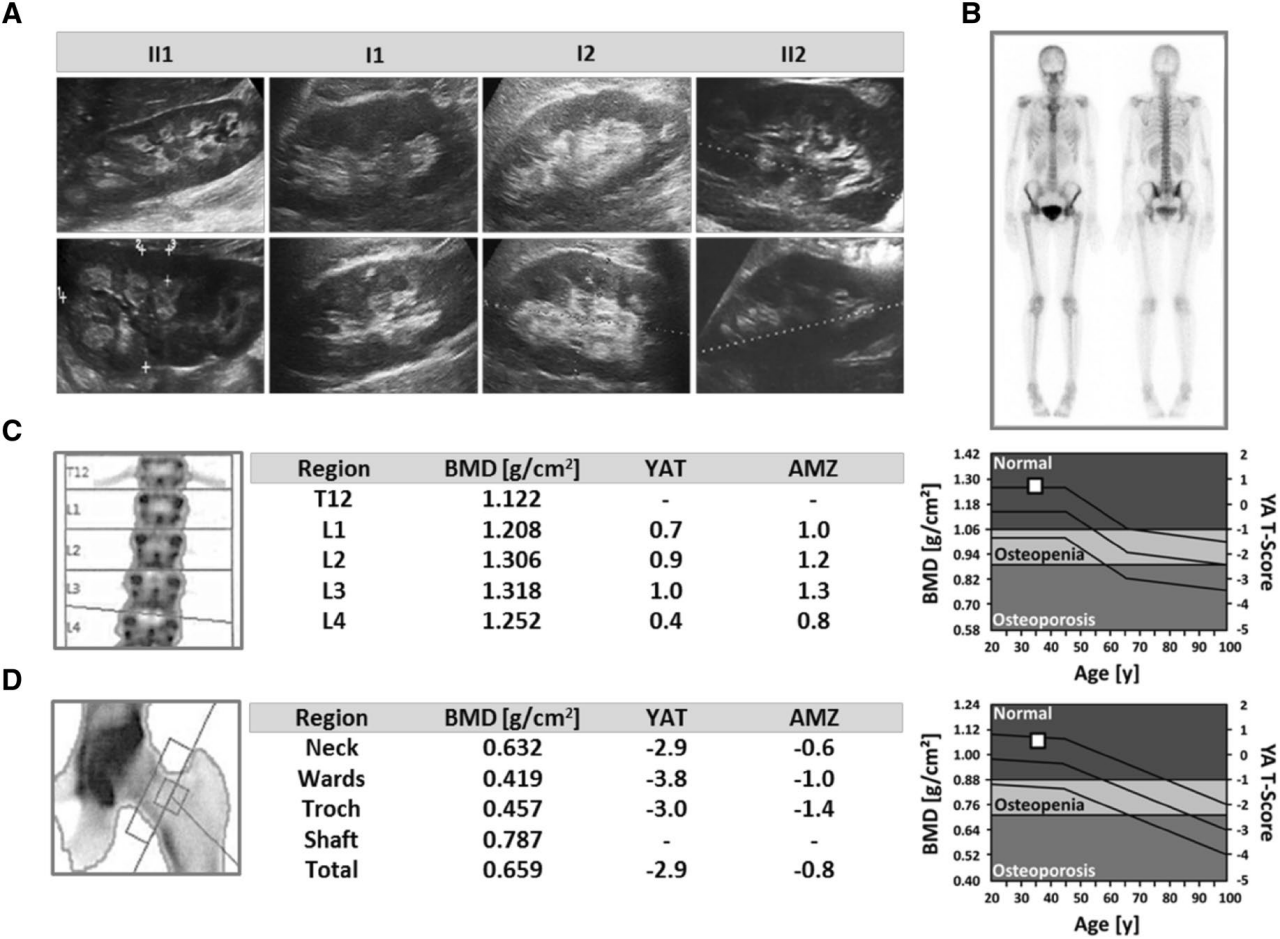
Clinical characteristic of the index family. **a** Renal ultrasound of the index patient (II1), her parents (I1—father, I2—mother), and her brother (II2). Bilateral corticomedullary renal calcifications indicating nephrocalcinosis is displayed in the index patient. Mild renal calcifications can be seen in both parents and the brother’s right kidney (II2). Upper panel—right kidney, lower panel—left kidney. **b** Bone scan of the index patient (II1) shows normal technetium uptake as sign of undisturbed bone metabolism. **c, d** Dual X-ray absorptiometry (DXA) of the index patient (II1) shows normal bone mineral density at both sites, spinal and femoral

**Fig. 2:**
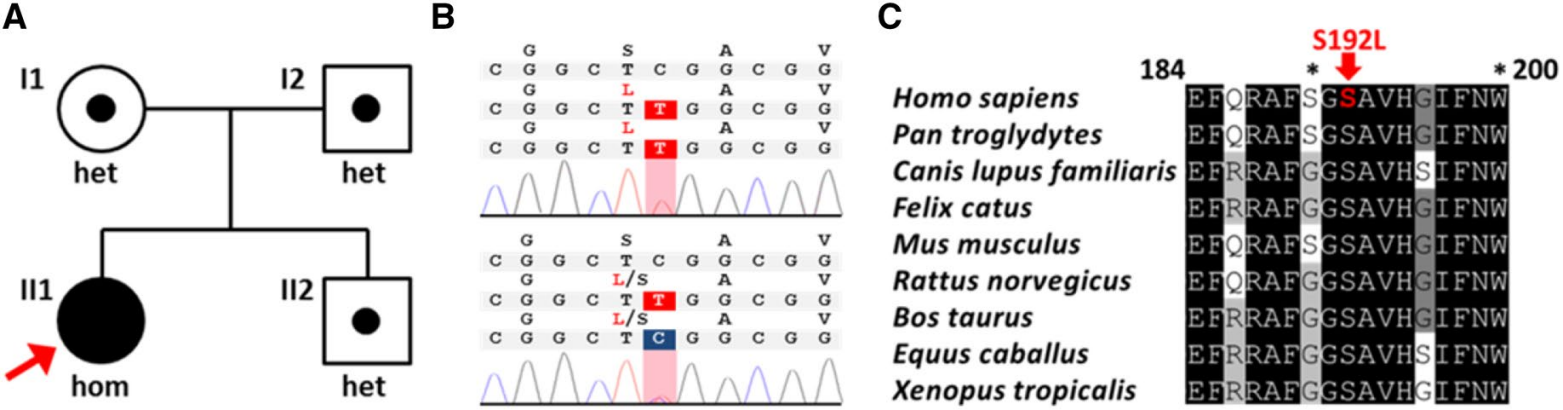
Mutation analysis of the index family. **a** Pedigree of the index family. The index patient is denoted by a red arrow. **b** Chromatogram of the index patient (II1) showing the homozgyous c.575C > T (*SLC34A3*) variant (NM_080877.2) above the heterozygous change, as present in the other family members (I1, I2, II2). **c** Evolutionary conservation of SLC34A3/NaPi2c at amino acid position Ser192 (in red) and neighboring residues (p. 184–200) according to NM_001177316

In a 32-year-old female (index patient, II1) with nephrocalcinosis on renal ultrasound scanning, we observed mild hypophosphatemia, hypercalcemic hypercalciuria, and reduced tubular phosphate reabsorption. Vitamin D3, PTH, and FGF23 levels were within the reference range (Table 1; Fig. 1a). The patient was of normal height (1.70 m) and weight (55 kg) (BMI 19.0 kg/m^2^) and did not report any prior kidney stone passages. Past medical history was negative for fractures or bone abnormalities and family history was completely negative for both, renal and bone disease. Upon targeted next-generation sequencing of known OMIM causes of nephrocalcinosis and nephrolithiasis (including *ATP6V1B1, ATP6V0A1, CLCN5, CLDN16, CLDN19, CYP24A1, PHEX, SLC34A3, SLC22A12, SLC2A9, SLC34A1, SLC3A1, SLC4A1, SLC7A9, SLC9A3R1*), we identified a homozygous c.575C>T, p.Ser192Leu mutation in *SLC34A3* as underlying genetic diagnosis (NM_001177316) (Fig. 2a, b). Segregation analysis confirmed heterozygous Ser192Leu variants in the parents (I1, I2) as well as in the younger brother (II2) of the index patient (Fig. 2a, b). As part of retrospective phenotyping, we undertook dual X-ray absorptiometry (DXA) and bone scan in the index patient. Both imaging techniques showed no abnormalities with regard to bone mineral density and technetium uptake, indicating normal bone metabolism (Fig. 1b–d). Further clinical assessment included biochemical analysis and renal ultrasound scanning of the heterozygous family members. Serum parameters revealed normal values; however, minimal signs of renal calcifications were seen in both parents and the brother (Table 1; Fig. 1a). In contrast to the only other previously reported individual (E/II-2) carrying a homozygous p.Ser192Leu mutation (Table 1), our index patient did not exhibit nephrolithiasis as a child and kidney function remained preserved despite renal calcifications. Upon daily oral phosphate supplementation (1800 mg/day), metabolic abnormalities of hypercalcemic hypercalciuria and hypophosphatemic hyperphosphaturia normalized completely within several weeks (Suppl. Table 1).

**Table 1:** Clinical characteristics of currently and previously reported SLC34A3-Ser192Leu patients

Individual	Sex/age	*SLC34A3* mutations/zygosity	Clinical phenotype	Laboratory parameters	References
Index	f/10	c.575C>T, p.Ser192Leu/hom	Nephrocalcinosis	Ca 2.52 mmol/l Pi 0.70 mmol/l AP 0.81 IU/l PTH 1.42 pmol/l 25(OH)D 78.3 nmol/l 1,25(OH)_2_D 136.5 pmol/l U-Ca/Crea 0.81 mmol/mmol Crea TRP 80%	**This study**
Brother of index	m/42	c.575C>T, p.Ser192Leu/het	Asymptomatic Mild renal calcifications	Ca 2.43 mmol/l Pi 1.05 mmol/l AP 1.31 IU/l U-Ca/Crea 1.11 mmol/mmol Crea TRP 74%	**This study**
Mother of index	f/71	c.575C>T, p.Ser192Leu/het	Asymptomatic	Ca 2.48 mmol/l Pi 0.87 mmol/l AP 1.38 IU/l U-Ca/Crea 0.34 mmol/mmol Crea TRP 96%	**This study**
Father of index	m/73	c.575C>T, p.Ser192Leu/het	Asymptomatic	Ca 2.39 mmol/l Pi 1.1 mmol/l AP 1.27 IU/l U-Ca/Crea 0.31 mmol/mmol Crea TRP 91%	**This study**
N137/CSS1355	f/67	c.575C>T, p.Ser192Leu/het	Kidney stones	Ca 2.58 mmol/l Pi 0.91 mmol/l AP 64 IU/l U-Ca/Crea 0.59 mmol/mmol Crea	**This study**
N77/CSS1162	f/65	c.575C>T, p.Ser192Leu/ het	Kidney stones	Ca 2.5 mmol/l Pi 1.15 mmol/l AP 109 IU/l U-Ca/Crea 0.05 mmol/mmol Crea	**This study**
5669	m/3	c.575C>T, p.Ser192Leu c.304 + 2T>C/ comp. het	HHRH with rickets	Ca 2.26 mmol/l Pi 0.87 mmol/l AP 908 IU/l 25(OH)D 35 nmol/l 1,25(OH)_2_D 335.4 pmol/l U-Ca/Crea 0.89 mmol/mmol Crea TRP 75%	[6]
E/II-2	m/6	c.575C>T, p.Ser192Leu/ hom	Kidney stones, nephrocalcinosis, low bone density	Ca 2.33 mmol/ Pi 1.1 mmol/l AP 966 IU/l PTH 0.63 pmol/l 25(OH)D 119 nmol/l 1,25(OH)_2_D 437 pmol/l U-Ca/Crea 1.03 mmol/mmol Crea TRP 107%	[12]
Case F (F/II-1)	f/6	c.575C>T, p.Ser192Leu c.1093+41_1094-15del (g.2615_2699del)/ comp. het	Pyelonephritis Nephrocalcinosis	Ca 2.57 mmol/l Pi 1.11 mmol/ PTH 1.38 pmol/l U-Ca 6–10 mg/Kg/day	[12]
E/I-1	f/53	c.575C > T, p.Ser192Leu/het	Kidney stones, nephrocalcinosis Hyperparathyroidism Thyro-parathyroidectomy	Ca 2.14 mmol/l Pi 1.1 mmol/l AP 240 IU/l PTH 1.27 pmol/l 25(OH)D 62 nmol/l 1,25(OH)_2_D 104 pmol/l U-Ca/Crea 0.50 mmol/mmol Crea TRP 90%	[12]
E/I-2	m/56	c.575C>T, p.Ser192Leu/het	No symptoms	Ca 2.22 mmol/l Pi 0.8 mmol/l PTH 3.92 pmol/l AP 210 IU/l 25(OH)D 63 nmol/l 1,25(OH)_2_D 146 pmol/l U-Ca/Crea 0.11 m mol/mmol Crea TRP 87%	[12]
G/II-1	f/11	c.575C>T, p.Ser192Leu c.367delC/comp. het	Kidney stones, nephrocalcinosis	Ca 2.63 mmol/l Pi 1.26 mmol/l PTH < 0.3 pmol/l 25(OH)D 45 nmol/l 1,25(OH)_2_D 283 pmol/l U-Ca/Crea 0.86 mmol/mmol Crea	[12]
Kindred D I-2	m/19	c.575C>T, p.Ser192Leu/het	Hypercalciuria, abnormal bone histology with increased rate of bone formation	Pi 0.74–0.89 mmol/l 1,25(OH)_2_D 96 pmol/l U-Ca/Crea 0.50 mmol/mmol Crea	[7]
Kindred D II-3	f/14	c.575C>T, p.Ser192Leu/het	HHRH mild bone disease	Pi 0.75–0.93 mmol/l 1,25(OH)_2_D 240 pmol/l U-Ca/Crea 0.57 mmol/mmol Crea TRP 80%	[7]
Kindred D II-4	f/11	c.575C>T, p.Ser192Leu/het	HHRH mild bone disease	Pi 0.75–0.91 mmol/l 1,25(OH)_2_D 344 pmol/l U-Ca/Crea 1.03 mmol/mmol Crea TRP 89%	[7]
Kindred D II-5	f/10	c.575C>T, p.Ser192Leu/het	HHRH severe rickets with bowing osteomalacia	Pi 0.77–1.13 mmol/l 1,25(OH)_2_D > 566 pmol/l U-Ca/Crea 0.79 mmol/mmol Crea TRP 87%	[7]

Age at the time of diagnosis/onset of symptoms

Patients identified in this study are highlighted bold

*Ca* calcium (normal 2.15–2.50 mmol/l), *Pi* phosphate (normal 0.84–1.45 mmol/l), *AP* alkaline phosphatase (normal 55–176 IU/l), *PTH* parathyroid hormone (normal 1.6–6.9 pmol/), *25(OH)D* 25-hydroxyvitamin D (normal 72–139 nmol/l), *1,25(OH)*_*2*_*D* 1,25-Dihydroxyvitamin D (normal 21.8-111.2 pmol/l), *U-Ca/Crea* urine calcium/creatine ratio (normal < 0.57 mmol/mmol Crea), *TRP* tubular reabsorption of phosphate (normal 82–90%)

In a next step, we sought to evaluate the frequency of p.Ser192Leu in a cohort of heterogeneous idiopathic adult kidney stone formers with or without hypercalciuria. Upon genetic analysis of 670 individuals with various types of kidney stones, we identified two more patients with heterozygous *SLC34A3* c.575C>T, p.Ser192Leu mutations. Both patients presented with a history of recurrent kidney stone events, but unspecific metabolic parameters and absence of renal calcification and overt bone manifestations (Table 1; N77/CSS1162 and N137/CSS1355).

**Fig. 3:**
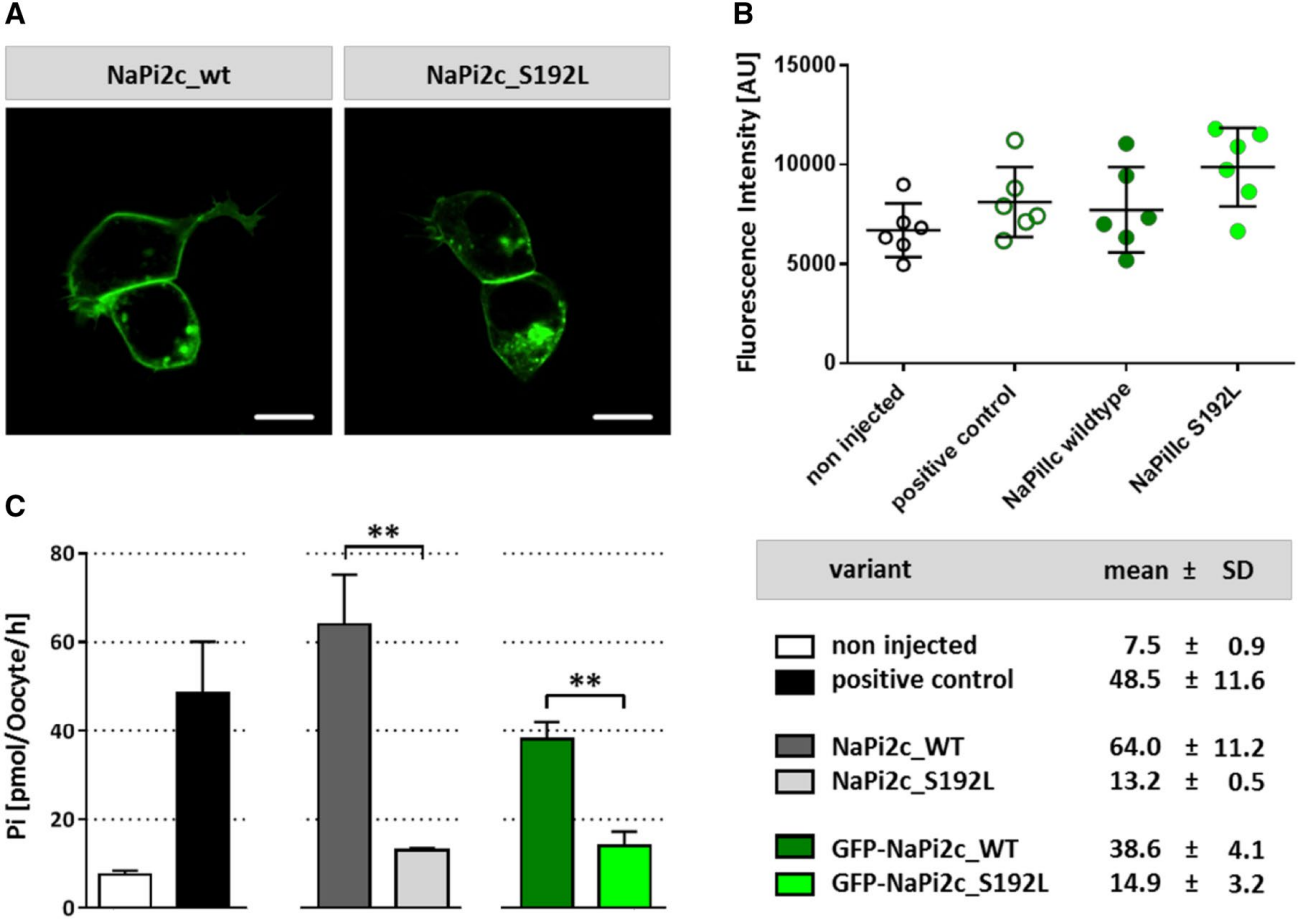
Functional evaluation of NaPi2c-Ser192Leu in comparison to wild type. **a** Plasma membrane localization of GFP-tagged mutant (Ser192Leu) and wild-type (WT)-NaPi2c proteins upon overexpression in HEK293 cells. **b** Fluorescence intensity, indicating plasma membrane localization, of negative control, positive control, NaPi2c mutant (Ser192Leu), and NaPi2c wild type (WT) upon *Xenopus* oocyte injection shows no significant difference. **c**^32^P_i_-uptake upon oocyte injection of NaPi2c mutant (Ser192Leu) is significantly disturbed in comparison to WT in both, GFP-tagged and non-GFP tagged conditions

To investigate the functional impact of the NaPi2c p.Ser192Leu mutation on a cellular level, we first performed overexpression of GFP-tagged mutant (Ser192Leu) and wild-type (WT) NaPi2c in HEK293 cells. While both constructs correctly localized to the plasma membrane, cells transfected with the mutant construct showed a slightly reduced expression on the cell surface in comparison to the wild type (Fig. 3a). Similarly, we injected both constructs into *Xenopus laevis* oocytes to test for membrane localization and functional *P*_i_ uptake. In line with the expression in HEK293, the mutant transporter was efficiently sorted to the plasma membrane (Fig. 3b). Upon oocyte ^32^P_i_-uptake, injected GFP and non-GFP mutants showed a significant reduction in transport activity in comparison to wild-type RNA (Fig. 3c). On the other hand, co-injection of WT and mutant RNA did not lead to a significant reduction of ^32^P_i_-uptake. To conclude, the p.Ser192Leu mutation does not have a significant impact on membrane sorting of NaPi2c but severely reduces its transport activity.

## Discussion

Evaluating the pathogenicity of genetic variants is crucial in the era of accelerated genetic diagnostics. To date, only a minority of the overall 38 reported HHRH-associated *SLC34A3* mutations (HGMD^Ⓡ^ professional version 2018.3) have been studied functionally [7, 10, 13]. Although p.Ser192Leu is one of the few repetitive findings in HHRH patients, it has never been assessed for its pathogenicity on a cellular level.

**Fig. 4:**
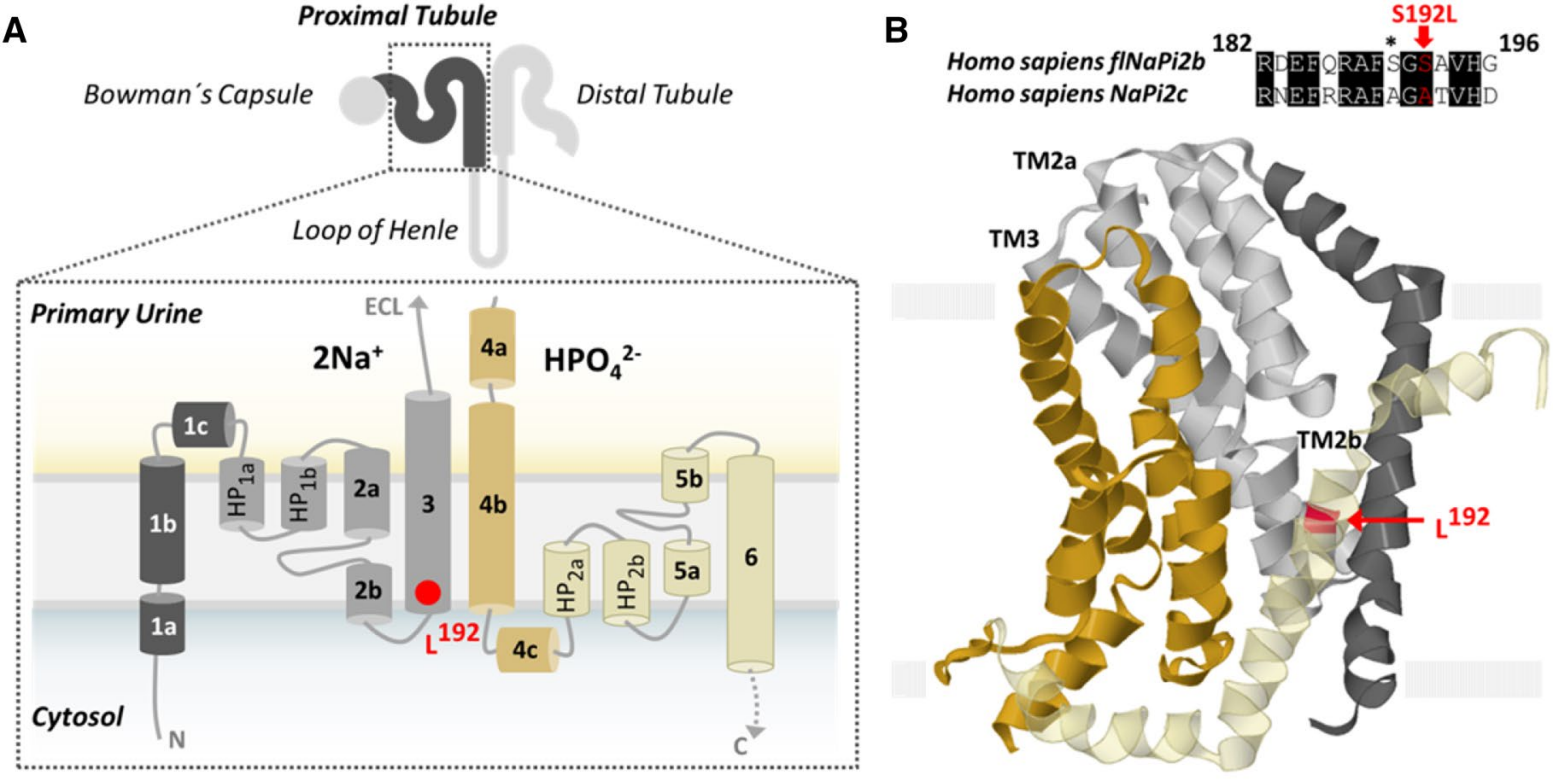
Localization and predicted topology of SLC34A3/NaPi2c wild type and mutant. **a** Cartoon of NaPi2c with its renal localization at the brush border of promixal tubules. The hypothetical structure based on sequence similarity with NaPi2b consists of six transmembrane domains (TM), where Ser192 (red) is part of the third transmembrane helix (light gray), adjacent to the substrate binding site. **b** Model of flNaPi2b (PM0080462) [15] showing NaPi2c Ser192 (red) (corresponding to NaPi2b Ala192) localization within the third transmembrane (TM) helix (light gray)

Based on its high sequence similarity with NaPi2b, the electroneutral P_i_ transporter NaPi2c is assumed to harbor six transmembrane domains [14] and two inverted core domains that move during the translocation process. Ser192 represents a highly conserved amino acid, most likely located within the third transmembrane domain adjacent to the substrate binding site (Figs. 2c, 4) [15, 16]. Human Ser192 is part of the previously described SSG/AAD motif (p.Ser189Ala, p.Ser191Ala, and p.Gly195Asp; corresponding to murine NaPi sequences) that is involved in binding of the first Na^+^ ion and affects the electrogenicity of co-transporter function [17]. A change of the adjacent Gly196 residue to arginine was also shown to result in trafficking defects with endoplasmatic reticulum retention and absence of membrane localization [13, 17]. Interestingly, unlike p.Gly196Arg, p.Ser192Leu seems to be adequately processed on its way to the cell surface, but lacks transport activity when expressed at the luminal brush border.

P.Ser192Leu occurs in the general population as a rare genetic variant in about 0.05% of individuals worldwide. With an allele frequency of 0.1%, p.Ser192Leu is reported almost exclusively in individuals of European, non-Finnish descent (86/87.762). Homozygosity, however, is not listed in SNP databases of the general population (ExAc/gnomAD). Although statistically not significant, our finding of two additional heterozygous p.Ser192Leu carriers in a population of 670 kidney stone formers suggests that p.Ser192Leu may be enriched in common nephrolithiasis (0.3%). This is in line with genome-wide association studies (GWAS) that reported a contribution of genetic variants within Mendelian nephrolithiasis genes to general kidney stone susceptibility [4]. Recently, Dasgupta et al. reported a significantly increased risk of renal calcification not only in patients with biallelic mutations, but also among heterozygous *SLC34A3* mutation carriers. In our functional approach to test for pathogenicity of heterozygous p.Ser192Leu, we did not identify a clear dominant influence of the mutated transporter on wild-type protein function. Similarly, in the family presented here, heterozygous carriers also showed no overt clinical manifestation, but slight biochemical abnormalities. These findings indicate a very mild effect of heterozygous mutations that may only become symptomatic with additional disease triggers, such as dehydration, lithogenic diet or a particular genetic background. Homozygosity of p.Ser192Leu in our patient is also associated with a very mild clinical phenotype without impairment of renal function and bone manifestations. Therefore, p.Ser192Leu, although clearly pathogenic upon expression studies, may be clinically compensated in some patients.

In summary, we report for the first time the deleterious effect of NaPi2cp.Ser192Leu by clinical presentation of a patient homozygous for the mutation and demonstrate its negative impact on *P*_i_ transport activity despite adequate membrane localization. In contrast to the evident molecular pathogenicity, the clinical picture of p.Ser192Leu may appear relatively mild and might therefore be overlooked in clinical practice. However, as demonstrated by the slightly differential phenotype of the only other reported individual with a homozygous p.Ser192Leu mutation (E/II-2—Table 1), clinical appearance is obviously influenced by individual environmental and/or genetic modifiers.

## Electronic supplementary material

Below is the link to the electronic supplementary material.

Supplementary material 1 (DOCX 22 KB)

## References

[1] Chande S, Bergwitz C (2018). Role of phosphate sensing in bone and mineral metabolism. Nat Rev Endocrinol.

[2] Wagner CA, Rubio-Aliaga I, Hernando N (2017). Renal phosphate handling and inherited disorders of phosphate reabsorption. An update. Pediatr Nephrol.

[3] Sayer JA (2017). Progress in understanding the genetics of calcium-containing nephrolithiasis. J Am Soc Nephrol JASN.

[4] Oddsson A, Sulem P, Helgason H, Edvardsson VO, Thorleifsson G, Sveinbjörnsson G, Haraldsdottir E, Eyjolfsson GI, Sigurdardottir O, Olafsson I et al. (2015). Common and rare variants associated with kidney stones and biochemical traits. Nat Commun 6:7975 10.1038/ncomms8975 PMC4557269 26272126

[5] Tieder M, Modai D, Shaked U, Samuel R, Arie R, Halabe A, Maor J, Weissgarten J, Averbukh Z, Cohen N (1987). “Idiopathic” hypercalciuria and hereditary hypophosphatemic rickets. Two phenotypical expressions of a common genetic defect. N Engl J Med.

[6] Lorenz-Depiereux B, Benet-Pages A, Eckstein G, Tenenbaum-Rakover Y, Wagenstaller J, Tiosano D, Gershoni-Baruch R, Albers N, Lichtner P, Schnabel D (2006). Hereditary hypophosphatemic rickets with hypercalciuria is caused by mutations in the sodium-phosphate cotransporter gene SLC34A3. Am J Hum Genet.

[7] Bergwitz C, Roslin NM, Tieder M, Loredo-Osti JC, Bastepe M, Abu-Zahra H, Frappier D, Burkett K, Carpenter TO, Anderson D (2006). SLC34A3 mutations in patients with hereditary hypophosphatemic rickets with hypercalciuria predict a key role for the sodium-phosphate cotransporter NaPi-IIc in maintaining phosphate homeostasis. Am J Hum Genet.

[8] Bergwitz C, Miyamoto K-I (2019). Hereditary hypophosphatemic rickets with hypercalciuria pathophysiology, clinical presentation, diagnosis and therapy. Pflugers Archiv Eur J Physiol.

[9] Halbritter J, Seidel A, Müller L, Schönauer R, Hoppe B (2018). Update on hereditary kidney stone disease and introduction of a new clinical patient registry in Germany. Front Pediatr.

[10] Jaureguiberry G, Carpenter TO, Forman S, Jüppner H, Bergwitz C (2008). A novel missense mutation in SLC34A3 that causes hereditary hypophosphatemic rickets with hypercalciuria in humans identifies threonine 137 as an important determinant of sodium-phosphate cotransport in NaPi-IIc. Am J Physiol Renal Physiol.

[11] Markovich D (2008). Expression cloning and radiotracer uptakes in *Xenopus laevis* oocytes. Nat Protoc.

[12] Dasgupta D, Wee MJ, Reyes M, Li Y, Simm PJ, Sharma A, Schlingmann K-P, Janner M, Biggin A, Lazier J (2014). Mutations in SLC34A3/NPT2c are associated with kidney stones and nephrocalcinosis. J Am Soc Nephrol JASN.

[13] Haito-Sugino S, Ito M, Ohi A, Shiozaki Y, Kangawa N, Nishiyama T, Aranami F, Sasaki S, Mori A, Kido S (2012). Processing and stability of type IIc sodium-dependent phosphate cotransporter mutations in patients with hereditary hypophosphatemic rickets with hypercalciuria. Am J Physiol Cell Physiol.

[14] Radanovic T, Gisler SM, Biber J, Murer H (2006). Topology of the type IIa Na+/P(i) cotransporter. J Membr Biol.

[15] Patti M, Fenollar-Ferrer C, Werner A, Forrest LR, Forster IC (2016). Cation interactions and membrane potential induce conformational changes in NaPi-IIb. Biophys J.

[16] Fenollar-Ferrer C, Forster IC, Patti M, Knoepfel T, Werner A, Forrest LR (2015). Identification of the first sodium binding site of the phosphate cotransporter NaPi-IIa (SLC34A1). Biophys J.

[17] Bacconi A, Virkki LV, Biber J, Murer H, Forster IC (2005). Renouncing electroneutrality is not free of charge. Switching on electrogenicity in a Na+-coupled phosphate cotransporter. Proc Natl Acad Sci USA.

