# Supplementary material for: Evaluating pathogenicity of SLC34A3-Ser192Leu, a frequent European missense variant in disorders of renal phosphate wasting
Source: Urolithiasis. 2019 Feb 23;47(6):511–9. doi: 10.1007/s00240-019-01116-2 (PMC6825645; doi:10.1007/s00240-019-01116-2)
Supplement: Supplementary file 1 — Supplementary material 1 (DOCX 22 KB) [file 240_2019_1116_MOESM1_ESM.docx]

**Suppl. Table 1**

|  | Visit 1  Month 1 | Visit 2  Month 3 | Visit 3  Month 4 | Visit 4  Month 7 | Visit 5  Month 12 | Visit 6  Month 18 |
| --- | --- | --- | --- | --- | --- | --- |
| S-Calcium  (mmol/l) | 2.52 (+) | 2.39 | 2.33 | 2.35 | 2.30 | 2.32 |
| S-Phosphate  (mmol/l) | 0.79 (-) | 0.75 (-) | 0.93 | 0.85 | 0.82 (-) | 0.94 |
| PTH  (pmol/l) | n.d. | 1.42 (-) | 1.99 | 1.42 (-) | 1.99 | 1.97 |
| U-Calcium  (mmol/mmol Crea) | 0.81 (+) | 0.49 | 0.12 | 0.33 | 0.23 | 0.50 |
